# Purification and Partial Structural Characterization of a Complement Fixating Polysaccharide from Rhizomes of *Ligusticum chuanxiong*

**DOI:** 10.3390/molecules22020287

**Published:** 2017-02-14

**Authors:** Yuan-Feng Zou, Yu-Ping Fu, Xing-Fu Chen, Ingvild Austarheim, Kari Tvete Inngjerdingen, Chao Huang, Lemlem Dugassa Eticha, Xu Song, Lixia Li, Bin Feng, Chang-Liang He, Zhong-Qiong Yin, Berit Smestad Paulsen

**Affiliations:** 1 Natural Medicine Research Center, College of Veterinary Medicine, Sichuan Agricultural University, Wenjiang 611130, China; yuanfengzou@sicau.edu.cn (Y.-F.Z.); yupingfu424@163.com (Y.-P.F.); huangchao@sicau.edu.cn (C.H.); tonysong838@aliyun.com (X.S.); lilixia905@163.com (L.L.); lorri190@126.com (C.-L.H.); yinzhongq@163.com (Z.-Q.Y.); 2 Key Laboratory of Crop Ecophysiology and Farming System in Southwest China, Ministry of Agriculture, College of Agronomy, Sichuan Agricultural University, Wenjiang 611130, China; 3 Department of Pharmaceutical Chemistry, School of Pharmacy, University of Oslo, P.O. Box 1068, Blindern 0316 Oslo, Norway; ingvild.austarheim@gmail.com (I.A.); k.t.inngjerdingen@farmasi.uio.no (K.T.I.); lemlemde@student.farmasi.uio.no (L.D.E.); b.s.paulsen@farmasi.uio.no (B.S.P.); 4 Animal Nutrition Institute, Sichuan Agricultural University, Wenjiang 611130, China; fengbin@sicau.edu.cn

**Keywords:** *Ligusticum chuanxiong*, polysaccharides, purification, pectic polysaccharide, complement system

## Abstract

Rhizome of *Ligusticum chuanxiong* is an effective medical plant, which has been extensively applied for centuries in migraine and cardiovascular diseases treatment in China. Polysaccharides from this plant have been shown to have interesting bioactivities, but previous studies have only been performed on the neutral polysaccharides. In this study, LCP-I-I, a pectic polysaccharide fraction, was obtained from the 100 °C water extracts of *L. chuangxiong* rhizomes and purified by diethylaminethyl (DEAE) sepharose anion exchange chromatography and gel filtration. Monosaccharide analysis and linkage determination in addition to Fourier transform infrared (FT-IR) spectrometer and Nuclear magnetic resonance (NMR) spectrum, indicated that LCP-I-I is a typical pectic polysaccharide, with homo-galacturonan and rhamnogalacturonan type I regions and arabinogalactan type I and type II (AG-I/AG-II) side chains. LCP-I-I exhibited potent complement fixation activity, ICH_50_ of 26.3 ± 2.2 µg/mL, and thus has potential as a natural immunomodulator.

## 1. Introduction

Chuanxiong Rhizoma (Chuanxiong, CX), the dried rhizome of *Ligusticum chuanxiong* Hort (Umbelliferae), is an effective medical plant, which has been extensively applied for centuries in migraine and cardiovascular diseases treatment in China [1]. Among ailments that have been treated by *L. chuanxiong* are various types of inflammatory disorders such as rheumatic arthritis, various types of pain such as headaches, chronic bronchitis, menstrual disorders, low blood circulation, angina pectoris, stroke and coronary heart disorders [2,3,4]. In addition, it has also been added to food as a prophylactic against illnesses [2]. Many reports indicate that the main components of *L. chuanxiong* are essential oil [5], alkaloids [6], phenolic acids [6,7], phthalides [6,8] and polysaccharides [2]. 

Polysaccharides isolated from *L. chuanxiong* have been shown to have several bioactivities, such as antioxidant activity [2,9], anticancer [10] and antibacterial activity [11,12]. Most of the reports on *L. chuanxiong* polysaccharides focus on the extraction techniques. These techniques include the following methods: microwave [13], pectinase treatment [14], cellulose enzyme treatment [15], ultrasonic-assisted extraction [11,16] and boiling water extraction [2]. Only a few polysaccharides from *L. chuanxiong* have been characterized, and they are all neutral polysaccharides. Basically, they are only related to monosaccharide compositions and molecular weight [2,17,18]. As mentioned above, polysaccharides have been shown to have effects on several biological systems. Thus, it was of interest to further study the structural properties and immunomodulating activity of polysaccharides from *L. chuanxiong*.

## 2. Results and Discussion

### 2.1. Extraction and Fractionation of Polysaccharide Fractions

**Figure 1:**
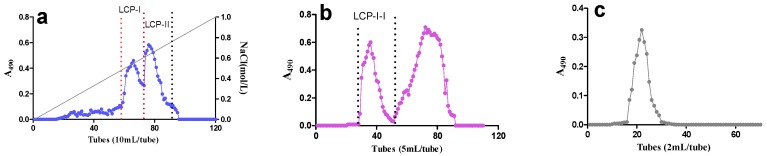
The carbohydrate elution profiles were monitored using the phenol–sulfuric acid assay (A490 is the absorbance at 490 nm). (**a**) Ion exchange chromatography elution profile of fraction *L. chuanxiong* polysaccharide (LCP); (**b**) Gel filtration elution profile of fraction LCP-I; (**c**) Size exclusion chromatography elution profile of fraction LCP-I-I. A490 stands for absorbance at 490 nm as described in phenol–sulfuric acid method.

The rhizomes of *L. chuanxiong* were further extracted with 100 °C distilled water after pre-extraction with 96% EtOH. The crude water extract *L. chuanxiong* polysaccharide (LCP) was applied onto an anion exchange chromatography column, and two active acidic fractions—LCP-I and LCP-II—were hereby obtained. LCP-I-I was purified from LCP-I by gel filtration (Figure 1), and is the most active fraction among fractions obtained. The yields for LCP-I and LCP-II were determined to be 40% and 20%, respectively. The yield of fraction LCP-I-I was determined to be 39%.

### 2.2. Complement Fixation Activity

**Figure 2:**
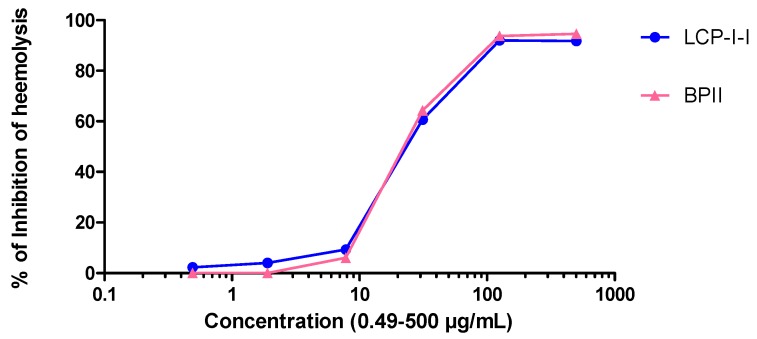
Complement fixating activities of purified polysaccharide fractions isolated from *L. crassicaulis* (LCP-I-I). Bioactive pectin from *B. petersianum* (BP-II) was used as a positive control.

The complement system is an important part of the innate immune system which also cooperates with the adaptive immune system in many ways [19]. The isolated polysaccharide fractions were tested for activity in the complement fixation assay. As can be seen from Figure 2, the purified polysaccharide fraction LCP-I-I showed potent human complement fixation activities in vitro. It showed comparable activity compared to the positive control BP-II (BPII, a highly active pectic polysaccharide from the aerial parts of *Biophytum petersianum* Klotzsch (syn. *B. umbraculum*)), as they gave similar ICH_50_ values (26.3 ± 2.2 µg/mL compared to 25.5 ± 2.5 µg/mL).

### 2.3. Chemical Composition of Polysaccharide LCP-I-I

The monosaccharide composition of the isolated active fraction LCP-I-I was determined by GC-analysis after methanolysis and trimethylsilylated (TMS) derivation. The monosaccharide composition present in LCP-I-I is typical for a pectic polysaccharide, consisting of galactose (Gal), galacturonic acid (GalA), arabinose (Ara) and rhamnose (Rha). As can be seen in Table 1, fraction LCP-I-I contains high amounts of total neutral monosaccharides (76.7%), and GalA is responsible for 22.6%. In addition, a minor amount of xylose (Xyl) is present. The monosaccharide composition of LCP-I-I is different from previous reports [2,17,18], as the reported polysaccharides that isolated *L. chuanxiong* were all neutral polysaccharides. This finding suggested that LCP-I-I could be considered as a novel polysaccharide isolated from rhizomes of *L. chuanxiong*.

The Bio-Rad protein and Folin–Ciocalteu assays showed that the polysaccharide fraction LCP-I-I did not contain protein or phenolic compounds. Size exclusion chromatography using dextran as standards was applied to determine the average molecular weight of the fraction obtained; the result indicated that the *Mw* of fraction LCP-I-I is 501.5 kDa.

### 2.4. Determination of Glycosidic Linkages in LCP-I-I

Linkage analysis of the active polysaccharide fraction LCP-I-I, was determined by Gas Chromatography-Mass Spectrometry (GC–MS) after the sample was subjected to reduction followed by methylation, hydrolysis, reduction, acetylation. The results obtained from linkage analysis are given in Table 2. Pectins are generally known to be composed of linear homo-galacturonan (HG) regions and branched rhamnogalacturonan (RG) I and II regions [20]. LCP-I-I contains 1,4 linked GalA moieties, which indicates a homo-galacturonan (HG) backbone. The presence of 1,4 linked GalA together with 1,2 linked Rha having branching on position 4 (1,2,4 linked Rha) suggested the presence of rhamnogalacturonan type I (RG-I) in LCP-I-I [21]. A high percentage of 1,4 linked Gal in LCP-I-I indicates the presence of arabinogalactan type I (AG-I) [22], and the presence of a small amount of AG-II polymer in LCP-I-I is suggested by the presence of Gal units being 1,3 linked; 1,6 linked and 1,3,6 linked Gal [23]. 

The Ara present in LCP-I-I is linked by terminal and 1,5; 1,3,5 and 1,2,3,5 indicate Ara present in LCP-I-I is a highly branched 1,5-arabinan. The methylation process might have resulted in under-methylation as there is a slightly higher degree of branch points than end groups. This may have been caused by a very compact molecule, or by low solubility in the solvent used for the methylation process. Xylose is one of the monosaccharides identified in small amounts after methanolysis. The presence of T-Glc and 1,4-linked Glc in LCP-I-I indicated the possible presence of glucan polymers originated from cellulose fragments. Contamination from starch, which possibly was not isolated from the samples, can also be a source of starch, but no starch was present in the sample as the iodine–potassium-iodide test was negative [24].

### 2.5. Other Structural Features

**Figure 3:**
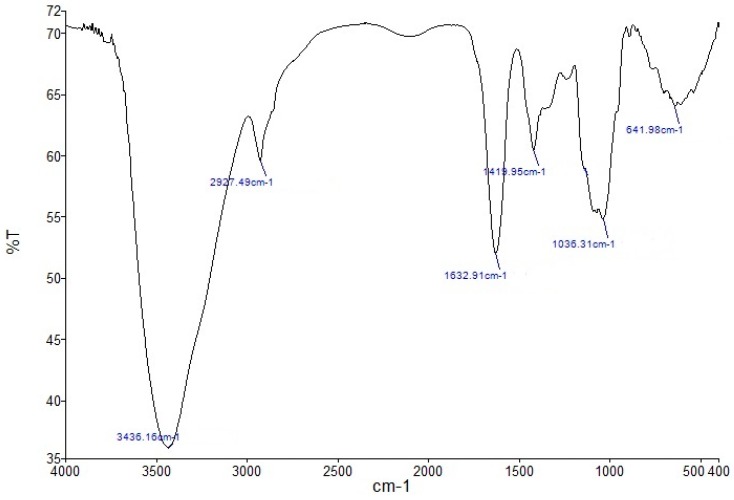
Fourier transform infrared spectroscopy of polysaccharide fraction LCP-I-I. “%T” stands for percentage of transmittance.

To characterize the polysaccharide fraction LCP-I-I, Fourier transform infrared (FT-IR) spectrometer (FT-IR) of the polysaccharide was performed in the range of 4000–400 cm^−1^. As shown in Figure 3, the FT-IR images of LCP-I-I gave characteristic absorptions of polysaccharides, such as peaks at 3436.16 cm^−1^, 2627.49 cm^−1^, 1632.91 cm^−1^, 1419.95 cm^−1^ and 1036.31 cm^−1^ [25,26,27,28]. The absence of 1250 cm^−1^ and 1735 cm^−1^ indicated that no methyl or acetyl esters were present in fraction LCP-I-I [29,30]. It was unusual to see that the hot water extraction gave a pectic polysaccharide not containing methyl and acetyl esters, however, there are some similar reports indicating that pectic polysaccharides isolated from hot water extracts do not contain any methyl or acetyl esters as confirmed by FT-IR and/or NMR spectra [31,32].

**Figure 4:**
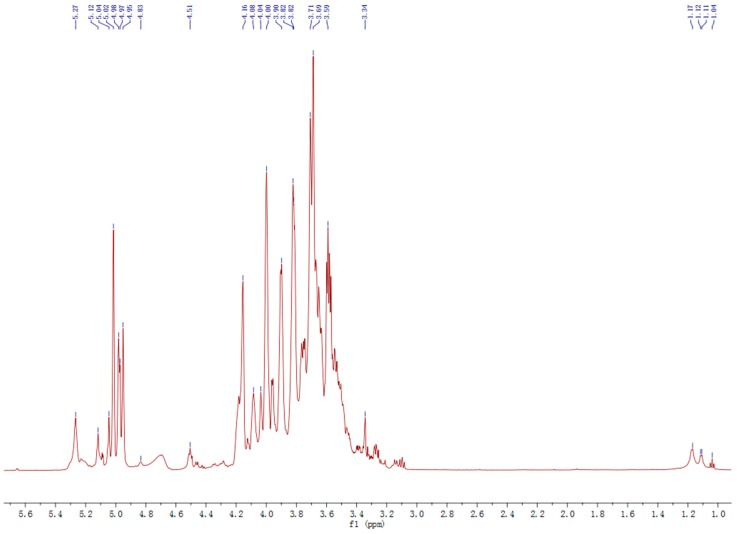
The ^1^H-NMR spectra of polysaccharide fraction LCP-I-I.

Polysaccharide fraction LCP-I-I was also characterized by one-dimensional (1D) NMR spectroscopy and chemical shift values were compared with data from literature [33,34,35,36]. Typically, the anomeric ^1^H signals of α-pyranoside are higher than 5 ppm while those of β-pyranoside are lower than 5 ppm [37]. The ^1^H-NMR spectrum of LCP-I-I contains eight main anomeric H at 5.27, 5.12, 5.04, 5.02, 4.98, 4.97, 4.95, 4.83 and 4.51 ppm, indicating the existence of both α- and β-configurations in the polysaccharide fraction LCP-I-I (Figure 4). The proton signal at 5.27 ppm indicated the presence ofα-L-Rha*p* in fraction LCP-I-I. The presence of signals at 1.12 and 1.17 ppm indicated the presence of methyl group of Rha in the fraction, but with low intensity. 

**Figure 5:**
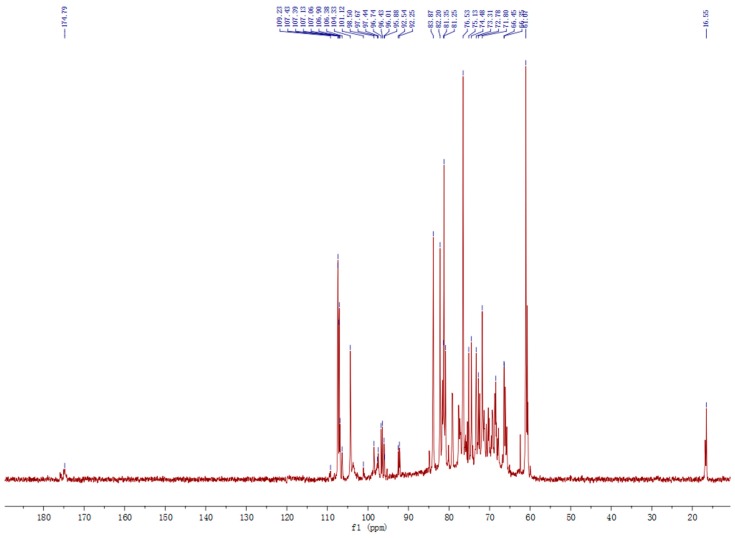
The ^13^C-NMR spectra of polysaccharide fraction LCP-I-I.

The ^13^C signals at 174.79 ppm corresponded to carbonyl carbon of unesterified α-1,4-GalpA, and the signal at 101.12 ppm corresponded to C-1 of α-1,4-Gal*p*A (Figure 5). Other signals such as 109.23 ppm, 107.29 ppm, 107.13 ppm, and 107.06 ppm corresponded to α-l-Ara*f*; 104.33 ppm correspond to β-d-Glc*p*; while 106.90 ppm and 106.38 ppm correspond to β-d-Gal*p*. The signals at 101.12 ppm and 16.55 ppm represented the anomeric carbon and C-6 of α-l-Rha [27]. These results suggested that fraction LCP-I-I is a typical pectic polysaccharide, with a HG region, RG-I region and arabinogalactan side chains. Pectins may bind calcium via carboxyl groups sitting on two separate pectin chains. This may be a strong binding so that the calcium is not removed by anion exchange chromatography [38]. This may lead to less movement in the molecule and could be the explanation for the low intensity of the signals around 175 ppm (Figure 5). The sample had a very low solubility, and because of this, it was difficult to obtain 2D NMR spectra that could be used for good interpretations of the shifts that should otherwise have been present.

## 3. Materials and Methods

### 3.1. Plant Material

The rhizomes of *L. chuanxiong* were harvested from Dujiangyan city, Sichuan Province, China, and identified by Xing-Fu Chen, College of Agronomy, Sichuan Agricultural University. A voucher specimen is deposited at the herbarium of College of Agronomy, Sichuan Agricultural University (Voucher no. 20150608). The rhizomes were washed, dried and pulverized to a fine powder using a mechanical grinder. 

### 3.2. Extraction of Polysaccharides

Two hundred grams of powdered rhizomes was first extracted twice with 96% ethanol (6 L) at 70 °C for 6 h until no color extracted in order to remove the lipophilic and low molecular weight compounds. The residue (163.2 g) was further extracted with 5 L of 100 °C distilled water two times for 2 h; the extracts were combined, concentrated, dialyzed at cut-off 3500 Da and lyophilized, and denominated as LCP (9.2 g). 

The crude extracts were first purified by anion exchange chromatography. The LCP (200 mg) was dissolved in 10 mL distilled water, filtered through 0.45 μm filters and applied to a diethylaminethyl (DEAE) Sepharose (Fast Flow, FF) column (Beijing Rui Da Heng Hui Science Technology Development Co., Ltd., Beijing, China). The neutral fractions were first eluted with 1.5 column volume (1 L) distilled water (at 2 mL/min), while the acidic fractions were eluted with a linear NaCl gradient in water (1.6 L, 0–1.5 M) at 2 mL/min. The carbohydrate elution profiles were monitored using the phenol–sulfuric acid assay [39]. The related fractions were pooled, dialyzed at cut-off 3500 Da against distilled water for removal of NaCl, concentrated and lyophilized. 

The acidic fractions were dissolved in elution buffer (10 mM NaCl), filtered through a Millipore filter (0.45 μm), and subjected to gel filtration after application on a Sepharose 6FF column (Beijing Rui Da Heng Hui Science Technology Development Co., Ltd., Beijing, China), and eluted with 10 mM·NaCl at 1.0 mL/min. Fractions were pooled based on the elution profile, as determined by the phenol–sulfuric acid assay, dialyzed and lyophilized.

### 3.3. Complement Fixation Assay

The complement system is an important part of the innate immune system which also cooperates with the adaptive immune system in many ways. Complement does, among other things, play a direct part in the defense, such as primary defense against bacterial invasions and viral infections. The complement fixation test is based on the inhibition of hemolysis of antibody-sensitized sheep red blood cells (SRBC) by human sera as described by Michaelsen et al. (Method A) [40]. BPII, a highly active pectic polysaccharide from the aerial parts of *Biophytum petersianum* Klotzsch (syn. *B. umbraculum*) [41], was used as a positive control. Inhibition of lysis induced by the test samples was calculated by the formula [(A_control_ − A_test_)/A_control_] × 100%. From these data, a dose–response curve was created to calculate the concentration of a test sample giving 50% inhibition of lysis (ICH_50_). A low ICH_50_ value means a high complement fixation activity. 

### 3.4. Chemical Compositions

The monosaccharide composition of the fraction with potent complement fixation activity was determined by gas chromatography of the trimethylsilylated (TMS) derivatives of the methyl-glycosides obtained after methanolysis with 3 M hydrochloric acid in anhydrous methanol for 24 h at 80 °C [42]. Mannitol was used as an internal standard. The TMS derivatives were analyzed by capillary gas chromatography on a Focus GC (Thermo Scientific, Milan, Italy). The injector temperature was 250 °C, the detector temperature 300 °C and the column temperature was 140°C when injected, then increased with 1 °C/min to170 °C, followed by 6 °C/min to 250 °C and then 30 °C/min to 300 °C. The total amount of phenolic compounds in the purified polysaccharide fractions were quantitatively determined using the Folin–Ciocalteu assay (Sigma-Aldrich, St. Louis, MO, USA) [43]. The protein content of the polysaccharide fractions was determined by the Bio-Rad protein assay (Bio-Rad, Hercules, CA, USA), based on the method of Bradford [44].

### 3.5. Linkage Determination

Glycosidic linkage elucidation was performed by GC–MS of the partly methylated alditol acetates. Prior to methylation, the activated uronic acids were reduced with NaBD_4_ to their corresponding neutral sugars. After reduction of the polymers, methylation, hydrolysis, reduction and acetylation [45] were carried out. The derivatives were analyzed by GC–MS using a GC–MS-QP2010 (Shimadzu, Kyoto, Japan) attached to a Restek Rxi-5MS column (30 m; 0.25 mm i.d.; 0.25 µm film; Restex, PA, USA). The injector temperature was 280 °C, the ion source temperature 200 °C and the interface temperature 300 °C. The column temperature was 80 °C when the sample was injected, then increased by 10 °C/min to 140 °C, followed by 4 °C/min to 210 °C and then 20 °C/min to 300 °C. Helium was the carrier gas (pressure control: 80 kPa). The compound at each peak was characterized by an interpretation of the retention times and the characteristic mass spectra. The estimation of the relative amounts of each linkage type was related to the total amount of each monosaccharide type as determined by methanolysis [46].

### 3.6. Molecular Weight Determination

The homogeneity and molecular weight of the native purified polysaccharide fraction was determined by size exclusion chromatography on a Hiload™ 16/60 Superdex™ 200 prep grade column (GE Healthcare, Uppsala, Sweden) combined with the Äkta system (FPLC, Pharmacia Äkta, Amersham Pharmacia Biotech, Uppsala, Sweden). Dextran polymers (Pharmacia, Uppsala, Sweden) of 10, 40, 70, 500 and 2000 kDa were used as calibration standards [47]. The phenol–sulfuric acid method was employed to determine the carbohydrate elution profiles of the polysaccharides fractions (Figure 1c).

### 3.7. FT-IR and NMR Spectroscopy

Approximately 1 mg of the polysaccharide sample was mixed with 150 mg of dried KBr powder, and pressed into a 1 mm thick disk for the analysis using a PerkinElmer FT-IR spectrophotometer (PerkinElmer, Waltham, MA, USA). The IR spectra were recorded in the range of 4000–400 cm^−^^1^ [48].

Polysaccharide fraction LCP-I-I was dissolved in D_2_O, deuterium-exchange three times by freeze-drying in D_2_O.^1^H-NMR and ^13^C-NMR spectra of LCP-I-I were recorded in D_2_O solution on a Bruker AV800 instrument (Bruker, Rheinstetten, Germany) at a temperature of 25 °C.

## 4. Conclusions

Polysaccharides isolated from the rhizomes of *L. chuanxiong* have been shown to exhibit several bioactivities, but no report about complement fixation activity has been published previously. The complement system plays a direct part in the immune defense system; therefore, the traditional use of this medicinal plant may be, at least partly, connected to the complement system. The polysaccharide obtained in the present study, LCP-I-I, was shown to be a pectic polysaccharide; the monosaccharide compositions and preliminary structure of LCP-I-I were different from previous studies. It contains HG and RG-I regions and AG-I/AG-II side chains, thus it could be considered as a novel polysaccharide isolated from rhizomes of *L. chuanxiong*. LCP-I-I exhibited potent complement fixation activity, and has potential as a natural immunomodulator.

## Figures and Tables

**Table 1:** Monosaccharide composition (mol %) and *Mw* (kDa) of polysaccharide fraction LCP-I-I obtained from rhizomes of *L. chuanxiong*.

	LCP-I-I
Ara ^a^	28.5
Rha ^a^	5.9
Xyl ^a^	0.6
Gal ^a^	26.3
Glc ^a^	15.4
GalA ^a^	22.6
*Mw* (kDa) ^b^	501.5

^a^ mol % related to the total content of the monosaccharides arabinose (Ara), rhamnose (Rha), xylose (Xyl), galactose (Gal), Glucose (Glc), and galacturonic acid (GalA). ^b^ The molecular weight (*Mw*) was determined by size exclusion chromatography.

**Table 2:** The linkage composition (mol %) of the monosaccharides present in polysaccharide fractions LCP-I-I obtained from rhizomes of *L. chuanxiong* determined by Gas Chromatography-Mass Spectrometry (GC–MS) after methylation.

Monosaccharide	Linkage Type	LCP-I-I
Ara	T*f*	9.4
	1→5*f*	4.7
	1→3,5*f*	11.1
	1→2,3,5*f*	3.4
Rha	T*p*	0.5
	1→2*p*	2.6
	1→2,4*p*	2.8
Gal	T*p*	4.9
	1→4*p*	14.1
	1→3*p*	2.4
	1→6*p*	1.2
	1→3,4*p*	1.2
	1→3,6*p*	2.4
Glc	T*p*	3.1
	1→4*p*	12.3
GalA	1→4*p*	22.6
